# Bayesian Model of Protein Primary Sequence for Secondary Structure Prediction

**DOI:** 10.1371/journal.pone.0109832

**Published:** 2014-10-14

**Authors:** Qiwei Li, David B. Dahl, Marina Vannucci, Hyun Joo, Jerry W. Tsai

**Affiliations:** 1 Department of Statistics, Rice University, Houston, Texas, United States of America; 2 Department of Statistics, Brigham Young University, Provo, Utah, United States of America; 3 Department of Chemistry, University of the Pacific, Stockton, California, United States of America; University of Michigan, United States of America

## Abstract

Determining the primary structure (i.e., amino acid sequence) of a protein has become cheaper, faster, and more accurate. Higher order protein structure provides insight into a protein’s function in the cell. Understanding a protein’s secondary structure is a first step towards this goal. Therefore, a number of computational prediction methods have been developed to predict secondary structure from just the primary amino acid sequence. The most successful methods use machine learning approaches that are quite accurate, but do not directly incorporate structural information. As a step towards improving secondary structure reduction given the primary structure, we propose a Bayesian model based on the knob-socket model of protein packing in secondary structure. The method considers the packing influence of residues on the secondary structure determination, including those packed close in space but distant in sequence. By performing an assessment of our method on 2 test sets we show how incorporation of multiple sequence alignment data, similarly to PSIPRED, provides balance and improves the accuracy of the predictions. Software implementing the methods is provided as a web application and a stand-alone implementation.

## Introduction

**Figure 1:**
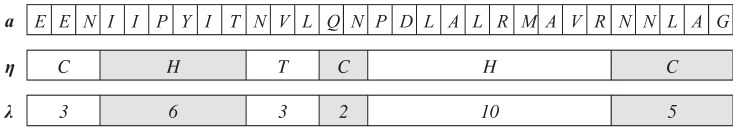
The first 29 amino acids from the protein clathrin 1*c*9*l* domain 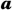 [46] with our associated parameterization 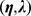 of the secondary structure. If this were the entire protein, then we would have *L* = 29, *M* = 6, and 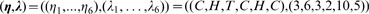.

For protein sequences of unknown structure and/or biological function, one of the first and quite insightful analyses of the linear sequence of amino acids (i.e., the primary structure) is a prediction of the secondary structure. Fig. 1 shows a four-state secondary structure definition of the primary amino acid sequence. Advances in genomic sequencing technologies have made obtaining protein sequences relatively cheap, accurate and fast, in comparison to the costly and involved approaches to solving a protein’s structure. However, the number of protein sequences far outpaces knowledge of their structure. Improvements in secondary structure prediction would have impact across many fields of computational biology. As the basis for higher order protein structure, more accurate secondary structure predictions is a necessary step for improved modeling of a protein’s fold [1], [2] and identification of its function [3]. Secondary structure modeling also plays an important role in the rational design of protein structure [4] and enzymatic function [5] as well as in drug development [6].

Depending on the set of protein sequences assessed, the accuracy of secondary structure prediction methods has improved steadily to an average of upwards of 80% [7]. The most successful approaches for secondary structure prediction apply machine learning algorithms to maximize the sequence relationship between a proteins’ primary sequences and their assigned secondary structure as defined by the program DSSP [8]. One of the early approaches that has become a standard in the field, PHD [9] and its current incarnation PredictProtein [10], employs an artificial neural network and sequence profiles in identifying secondary structure from a protein sequence. Other successful servers such as Jpred [11] and PSIPRED [12] also apply artificial neural networks. As an approach, neural net based prediction methods are quite popular and accurate [13], [14]. Other machine learning methods attempt to match prediction accuracy using hidden Markov models (HMM) [15]–[17] and support vector machines (SVM) [18], [19]. Due to their consistently high accuracy of prediction, these methods have become the *de facto* standard against which other secondary structure prediction methods measure their success, many of which have been evaluated in a recent review [20]. However, the accuracy has essentially remained at 80% for many years [2], [20].

Because these expert systems rely on indeterminate relationships between the primary sequence and a 3 or 4 state secondary structure classification, a potential approach to improving secondary structure predictions is to incorporate higher order structural information. The initial use of structural information to model protein secondary structure was based on the hydrophobic patterns found in amphipathic helices and sheets [21] or the hydrophilic spacing between residues and turn regions [22]. With the recent success of fragment-based structure prediction, numerous methods have incorporated structural information from local fragment prediction [23]–[25] to more global structural relationships [26]–[29] into secondary structure prediction. These recent methods have been able to reproduce the 80% accuracy of the machine learning approaches. The approach tested in this paper applies sequence to structure relationships defined by packing of residues based on the knob-socket model [30], [31].

**Figure 2:**
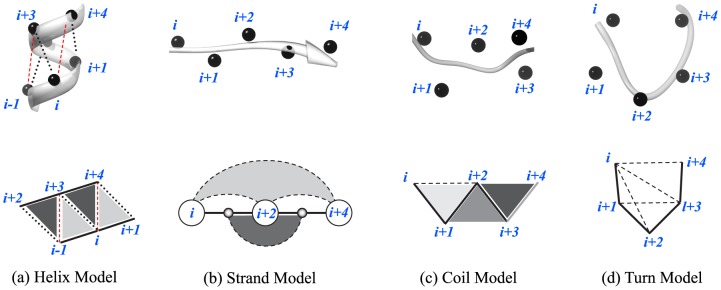
Local structural motifs used to model protein secondary structure as defined by the knob-socket model. On the top for each type of secondary structure, ribbon diagrams of the protein backbone with black spheres at C*α* positions are presented. On the bottom, two-dimensional lattice representations are shown of the local residue interactions that define secondary structure, where solid lines represent covalent contacts between residues and broken lines are packing interactions. Because only the local interactions are being considered to predict secondary structure, only the socket portion of the knob-socket model is used. The knob portion signifies interactions at the level of tertiary structure or packing of non-local residues distant in the protein sequence. Each of the 4 types of secondary structure are described in more detail. (a) Helix Model: Relative residue positions and interactions are shown. Two types of sockets are represented in different grey scale: 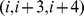 sockets in dark grey and 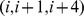 sockets in light grey. (b) Strand Model: Double-side sheet sockets are shown. Sockets 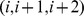 and 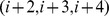 in white are facing one direction, a socket 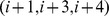 in dark grey faces the other side. Also, the side chain only socket 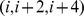 is shown in light grey. (c) Coil Model: Three types of coil sockets are shown. The socket 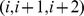 is closed socket with all three residues in contact one another, the socket 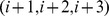 is open socket with 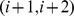 contact and 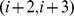 contact but no contact between 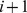 and 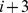, and the socket 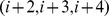 is strained socket with no contact between 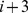 and 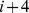. (d) Turn Model: Three residue sockets 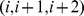, 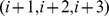, 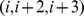, and 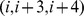 in the 5 residue turn are shown.

Improving the previous models of packing in helix [32] and sheet [33], the knob-socket model provides a simple and general motif to describe the packing in protein structure that has been shown to relate the primary sequence to the packing structure at both the secondary and tertiary structure levels in both helices [30] and sheets [31]. Whereas the previous knob-into-holes [32] and ridges-into-grooves [33] are each limited to describing packing at defined angles within only a single type of secondary structure, the knob-socket model encompasses all packing within proteins at all angles and between all types of secondary structure. The knob-socket model simplifies the convoluted packing of side-chains into regular patterns of a single knob residue from one element of secondary structure packing into a socket formed by 3 residues from another element of secondary structure. Because the composition of both the knobs and socket exhibit preferences for certain amino acids, this knob-socket model not only relates primary sequence to tertiary packing structure, but also associates the primary sequence with secondary structure packing. At the level of secondary structure, only the local 3-residue socket plays a role in this model, since the knob residue defines tertiary packing structure. The repetitive main-chain hydrogen bonding for regular secondary structure produces a consistent arrangement of sockets. The arrangements defines the secondary structure packing motifs that provide the sequence patterns to identify secondary structure (Fig. 2). This is the case even for the irregular coil secondary structure.

In this paper we propose a Bayesian model for secondary structure prediction given the primary structure. The method considers the packing influence of residues on the structure determination, including those packed close in space but distant in sequence. Fundamentally, secondary structure is defined by the regular hydrogen bonding patterns between the main-chain polar groups of amino acids in the linear sequence. From this definition, secondary structure is usually described by three classes (*α*-helix, *β*-sheets, and other or coil) for three-state predictions. The regular hydrogen bonding patterns define the states of *α*-helix or *β*-strand, whereas the irregular coil is defined by the lack of repetitive hydrogen bonding. Instead of hydrogen bond patterns, the knob-socket model identifies the regular patterns of packing not only in *α*-helices and *β*-strand, but also in coil structure (Fig. 2). We therefore develop a probabilistic model for secondary structure prediction that is informed by the packing structure between residue defined by the knob-socket model. The local sequence relationships are incorporated, similarly to PSIPRED [12] [34], as a set of multiple sequence alignments (MSA). The incorporation of MSA information has been a standard approach for many years [35]. We compare performances of our method with TorusDBN [36] and the benchmark machine learning approach PSIPRED, on two test sets. TorusDBN is a conformational sampling method (akin to a fragment library) and was not developed for secondary structure prediction. A side-benefit of the TorusDBN method, however, is the prediction of protein secondary structure. Our results demonstrate that adding local structural information (as defined by the knob-socket model) in the prior distribution increases our method’s accuracy to just below current standards on one of the test set and above on the larger test set.

We have developed both a web application and stand-alone implementation of the methods described in this paper. The website is http://bamboo.byu.edu and the stand-alone implementation is bamboo, an open-source R package available on the Comprehensive R Archive Network (CRAN). The package implements all the methods described in the paper and it provides all the data used in our analysis. Documentation and an example are provided. The package may be installed on the latest version of R by running: install.packages (“bamboo”).

## Results and Discussion

We evaluate the performance of our proposed method against the 3,344 chains in the ASTRAL30 and the 203 chains in the CASP9 data sets. For each data set, the DSSP annotation [8] was used as the true secondary structure state. Over the thousands of proteins, the secondary structure prediction based on the amino acid sequence 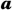 is compared to the DSSP value at each position. Since test sets were not part of the 15,470 chain training set, the accuracy reported for our approach compared to the true secondary structure value are reliable assessments of performance. The knob-socket packing was implemented in 2 different ways with 2 posterior summaries, which amounts to 4 separate methods. We compare the prediction accuracy of our method against 2 other secondary structure prediction methods: TorusDBN [36] and PSIPRED [12]. PSIPRED was run using 2 sequence databases: sequences from the PDB for PSIPRED-PDB and a non-redundant sequence database for PSIPRED-NR, described in more detail in the methods. The purpose of the PSIPRED-PDB is to provide a more direct comparison of methods, since the MSA’s used by our method were limited to sequences from the PDB [37]. However, with a deep sequence alignment as an input, PSIPRED-NR produces the best results, which coincide with the benchmark for secondary structure prediction accuracy.

Table 1 reports the percent accuracy of each method on the ASTRAL30 and CASP9 test sets in terms of classification recall, i.e., the percent of the true DSSP defined secondary states that are correctly predicted for 3 classes, or Q3. Because TorusDBN and PSIPRED are three-state models that do not predict turn residues, the turn predictions of our model were merged into the coil class to facilitate comparisons. TorusDBN and PSIPRED both performed consistently between the 2 test sets. Although secondary structure prediction is not the focus of TorusDBN, TorusDBN exhibits a high of 62% accuracy for the ASTRAL30 test set. For PSIPRED, using only sequences from solved structures performs up to 15 percentage points better than TorusDBN, while allowing deeper sequence alignments into the larger non-redundant sequence database increases the Q3 accuracy upwards of 20 points to the established standard of 80% on the ASTRAL30 data set and slightly higher 81% on the CASP9 data set. As a baseline for our method, the NonInfo-MAP uses a non-informative prior and maximization of the posterior probability, producing the lowest measured accuracy of 52% on the CASP9 data set. Implementing the secondary structure guided block sampling of the posterior distribution (NonInfo-MP in Table 1) provides a modest increase in prediction accuracy, although still a bit below TorusDBN for both test sets. The fact that TorusDBN and our NonInfo-MP essentially perform equally is to be expected given the fact that neither use an informative prior. Incorporating MSA information to inform our method’s prior distribution results in a significant improvement in accuracy, especially with the ASTRAL30 test set where both implementations of the posterior sampling reach accuracies above the PSIPRED-NR benchmark of 80% at 84% for MSA-MAP and 88% for MSA-MP. Accuracies improve for the CASP9 test set too, although they remain lower than PSIPRED-NR at 69% for MSA-MAP and 75% for MSA-MP. This decrease in performance is likely due to the different amount of MSA information available for the two data sets. For ASTRAL30, in fact, there were 201 sequences without an MSA, which is 6%, while for CASP9, there were 109 sequences, which is 54%.

**Table 1:** Overall Q3 accuracy (%) of TorusDBN, PSIPRED, and our method under different priors and segmentations on ASTRAL30 and CASP9 test data sets.

	Other methods			Our method			
Dataset	TorusDBN	PSIPRED-PDB	PSIPRED-NR	NonInfo-MAP	NonInfo-MP	MSA-MAP	MSA-MP
ASTRAL30	62	77	80	53	60	84	88
CASP9	61	75	81	52	58	69	74

Recall and precision is broken down for each of the secondary structure states to provide a more detailed understanding of Q3 prediction accuracy. For the ASTRAL30 and CASP9 sets respectively, a comparison of Q3 classification recall is shown in Tables 2 and 3 and Q3 classification precision is shown in Tables 4 and 5. The recall tables indicate that TorusDBN best predictions are of the helix and coil states. The majority of incorrectly predicted helix residues are assigned the coil state and the converse is true for the coil state. The strand residues are assigned essentially by chance with a uniform distribution over the 3 states. For precision, the TorusDBN results are consistently the same across all three states, where the distribution of prediction is 60% correct and then about equally mispredicted at 20% for the other 2 states. The PSIPRED program’s recall in both implementations and across both test sets performs the best at identifying the coil state, then the helix state and finally the strand state. Incorrect predictions of state are consistently coil that should be strand or helix. PSIPRED does not mix up helix and strand states often. Precision of PSIPRED predictions is the best for the helix state and worst for the coil state, where the strand state is twice as likely to be predicted as coil than the helix state. Reiterating the recall results, the precision of PSIPRED helix and strand predictions consistently are incorrectly assigned coil states.

**Table 2:** Classification Q3 recall (%) of TorusDBN, PSIPRED, and our method under different priors on ASTRAL30 test dataset.

	Other Methods									
	(a) TorusDBN			(b) PSIPRED-PDB				(c) PSIPRED-NR		
	Helix	Strand	Coil	Helix	Strand	Coil	Helix		Strand	Coil
Helix	69	30	23	77	5	9	79		3	6
Strand	7	33	6	3	66	8	1		68	6
Coil	24	37	71	20	29	83	20		29	88
Overall	62			77			80			

	Our Methods											
	(d) NonInfo-MAP			(e) NonInfo-MP			(f) MSA-MAP			(g) MSA-MP		
	Helix	Strand	Coil	Helix	Strand	Coil	Helix	Strand	Coil	Helix	Strand	Coil
Helix	42	16	17	50	12	13	84	2	8	90	1	6
Strand	17	43	14	15	51	11	1	79	6	1	85	5
Coil	41	41	69	35	37	76	15	19	86	9	14	89
Overall	53			60			84			88		

^*^Each column of the matrix represents the instances in an actual class, while each row represents the instances in a predicted class. Note that the sum of elements of each column equals to 100.

**Table 3:** Classification Q3 recall (%) of TorusDBN, PSIPRED, and our method under different priors on CASP9 test dataset.

	Other Methods								
	(a) TorusDBN			(b) PSIPRED-PDB			(c) PSIPRED-NR		
	Helix	Strand	Coil	Helix	Strand	Coil	Helix	Strand	Coil
Helix	72	31	24	77	6	10	82	2	7
Strand	6	30	5	4	62	10	1	69	7
Coil	22	39	71	19	32	80	17	29	86
Overall	61			75			81		

	Our Methods											
	(d) NonInfo-MAP			(e) NonInfo-MP			(f) MSA-MAP			(g) MSA-MP		
	Helix	Strand	Coil	Helix	Strand	Coil	Helix	Strand	Coil	Helix	Strand	Coil
Helix	41	16	18	47	14	14	66	8	13	73	7	11
Strand	18	42	14	16	49	12	7	61	10	7	67	9
Coil	41	42	68	37	37	74	27	31	77	20	26	81
Overall	52			58			69			74		

^*^Each column of the matrix represents the instances in an actual class, while each row represents the instances in a predicted class. Note that the sum of elements of each column equals to 100.

**Table 4:** Classification Q3 precision (%) of TorusDBN, PSIPRED, and our method under different priors on ASTRAL30 test dataset.

	Other Methods								
	(a) TorusDBN			(b) PSIPRED-PDB			(c) PSIPRED-NR		
	Helix	Strand	Coil	Helix	Strand	Coil	Helix	Strand	Coil
Helix	63	16	21	86	3	11	91	2	7
Strand	22	59	19	5	78	17	3	84	13
Coil	20	18	62	17	13	70	16	13	71
Overall	62			77			80		

	Our Methods											
	(d) NonInfo-MAP			(e) NonInfo-MP			(f) MSA-MAP			(g) MSA-MP		
	Helix	Strand	Coil	Helix	Strand	Coil	Helix	Strand	Coil	Helix	Strand	Coil
Helix	61	13	26	71	10	19	91	1	8	93	1	6
Strand	30	44	26	27	52	21	2	86	12	1	89	10
Coil	30	17	53	26	16	58	13	9	78	8	7	85
Overall	53			60			84			88		

^*^Each column of the matrix represents the instances in an actual class, while each row represents the instances in a predicted class. Note that the sum of elements of each row equals to 100.

**Table 5:** Classification Q3 precision (%) of TorusDBN, PSIPRED, and our method under different priors on CASP9 test dataset.

	Other Methods								
	(a) TorusDBN			(b) PSIPRED-PDB			(c) PSIPRED-NR		
	Helix	Strand	Coil	Helix	Strand	Coil	Helix	Strand	Coil
Helix	61	17	22	84	4	12	91	2	7
Strand	19	63	18	7	74	19	2	84	14
Coil	19	21	60	16	16	68	13	14	73
Overall	61			75			81		

	Our Methods											
	(d) NonInfo-MAP			(e) NonInfo-MP			(f) MSA-MAP			(g) MSA-MP		
	Helix	Strand	Coil	Helix	Strand	Coil	Helix	Strand	Coil	Helix	Strand	Coil
Helix	58	15	27	67	12	21	77	6	17	82	5	13
Strand	30	45	25	27	52	21	12	69	19	11	73	16
Coil	30	19	51	27	17	56	22	16	62	17	14	69
Overall	52			58			69			74		

^*^Each column of the matrix represents the instances in an actual class, while each row represents the instances in a predicted class. Note that the sum of elements of each row equals to 100.

For the baseline NonInfo-MP implementation of our method, the recall results (Tables 2 and 3) indicate that inclusion of this model for coil correctly predicts a high of 75% of coil residues. This result is accomplished by over-predicting the coil state such that coil is the major error in predicting helix and strand at around 37% for each. The precision for NonInfo-MP reveals that the helix state is most precisely predicted followed by coil and then sheet for both the ASTRAL30 (Table 4) and CASP9 (Table 5) test sets. NonInfo-MP and TorusDBN do not use prior information and, therefore, do not perform particularly well, our MSA-MP predictions perform very well on the ASTRAL30 set at 88% accuracy. The recall in Table 2 is at 90% for helix, 89% for coil and 85% for strand. The predominate error is to assign coil to helix and sheet at 9% and 14%, respectively. This is especially encouraging as the MSA-MP uses essentially the same sequence database as PSIPRED-PDB. The precision for the MSA-MP method (Table 3) corroborates these results. While TorsuDBN and PSIPRED accuracies are consistent between the 2 test sets, our MSA-MP method exhibits worse Q3 prediction accuracies for the CASP9 data set. However, the overall accuracy of 74% is on par with the PSIPRED-PDB value of 75%. For recall, the drop in accuracy accompanies an increase in incorrect assignment of helix and strands to coil. The precision drop with the MSA-MP on the CASP9 data set shows an increase in all of the off-diagonal misprediction states. Indeed, the NonInfo implementation of our method is used for those sequences without MSA information, resulting in the overall predictions being influenced by the over prediction of the coil state.

**Figure 3:**
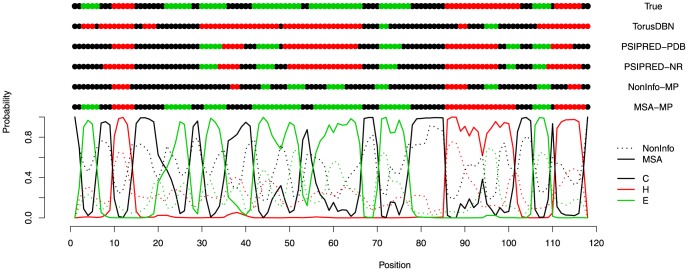
Marginal probability (MP) curves across positions for the phospholipase protein 3*rvc* [38]. Shown at the top is the true secondary structure, TorusDBN’s prediction, PSIPREDs’ prediction, and the prediction from our method (MP-MSA).

Fig. 3 compares the Q3 results from the different methods to the DSSP [8] defined states for the phospholipase protein 3*rvc* [38], where the marginal posterior probabilities from our NonInfo-MP and MSA-MP methods are also plotted. Performing the worst in this set are TorusDBN and our NonInfo-MP, and both of these are limited to primarily local information. As indicated in the recall and precision tables, TorusDBN over predicts the helix state and under predicts the sheet state, which is clearly shown by the prediction in Fig. 3. Our NonInfo-MP method is slightly better at finding regions of correct secondary structure, but the length and limits of secondary structure states are poorly predicted. As shown by Fig. 2, the longest sequence distance that the knob-socket model considers is 5 residues. Clearly, information limited to local residues is unable to accurately reproduce the native secondary structure over large segments of sequence. Yet, over all of our predictions using the NonInfo-MP implementation, the average difference between the incorrect and correct probabilities was 0.244, with a standard deviation of 0.173 and an interquartile range (that is, the difference between the 25*th* and the 75*th* percentiles) of 0.244. This implies that the probabilities are somewhat close in areas of misprediction. With a residue window of 15 residues [34], PSIPRED in both of its applications is able to better identify the sheet residues and define the transitions between the different secondary structure states. Adding in the MSA information, the MSA-MP method adds more global secondary structure state information to the local model provided but the knob-socket model. The accuracy in identification of secondary structure states is very accurate, with most of the errors in defining the ends of secondary structure segments. In the plot of the MSA-MP, the marginal probability for a certain type of structure is clearly dominant at many positions in the middle of secondary structure segments, with values close to 1, but drops at the residues that transition between secondary structure states, with values of 0.5 or a little higher. While the case shown in Fig. 3 displays a favorable prediction for our MSA-MP method, it is at these transition points where the marginal probabilities of the secondary structure states clearly show less confidence in the prediction. Plots of this type help us understanding how incorporating information about the global influences on secondary structure can aid prediction.

As noted above and shown in lower portion of Fig. 3, the posterior distribution of marginal probabilities facilitates inference and adds a level of interpretation available only through a Bayesian approach. In particular, these marginal probabilities provide an extra level of confidence in the predictions. The posterior distributions in Fig. 3 are not only higher in areas that are correctly predicted, but there is a great spread between the marginal probability of the correct state than the next closest incorrect state. For the transitions areas, the marginal probabilities are much closer in value. To quantify of this, the difference in probability between the correct and highest incorrect state have been calculated and are reported for each state in Table 6. In the ASTRAL30 data set, the difference is between 56% and 70% when the prediction is correct, whereas the difference is only between 34% and 39% when incorrect. In the CASP9 data set, the values are smaller in both cases, where the difference for correct predictions ranges from 42% to 54% and the difference for incorrect predictions is 24% to 28%. So, as a simple rule of thumb, a separation of over 50% would strongly indicate a good prediction, and values less than 50% would indicate that the prediction is potentially wrong.

**Table 6:** Means and standard deviations (in parenthesis) of differences in marginal probability between correctly predicted secondary structure (Correct) and the next highest probability, and between secondary structure predicted incorrectly (Wrong) and highest probability for ASTRAL30 and CASP9 data sets.

		Helix	Strand	Turn	Coil
ASTRAL30	Correct	0.71(0.25)	0.70(0.27)	0.59(0.29)	0.56(0.31)
	Wrong	0.34(0.26)	0.36(0.25)	0.39(0.27)	0.35(0.26)
CASP9	Correct	0.54(0.30)	0.53(0.30)	0.42(0.28)	0.45(0.34)
	Wrong	0.24(0.19)	0.25(0.19)	0.28(0.23)	0.28(0.22)

**Figure 4:**
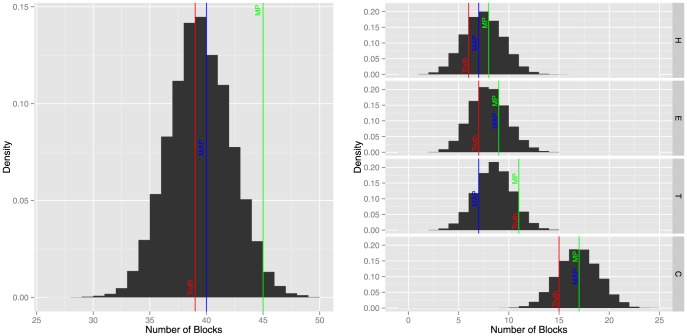
The posterior distribution of the number of blocks in total (left) and the number of blocks of each type (right) for protein T0622-D10 from the CASP9 data set. Also displayed is the number of the blocks in the truth, the MAP estimate, and the MP estimate in red, blue, and green color, respectively.

Not only can we examine the marginal posterior probability at each position, but our method allows us to make inference on the number of blocks in total and the number of blocks of each type. Take, for example, protein T0622-D10 from the CASP9 data set. These posterior distributions are plotted in Fig. 4. For this protein, the model estimated well the total number of blocks, but has over estimated the number of coil blocks and under estimated the number of turn blocks.

Our results reinforce the general concept that more context is necessary to understand the environment that induces secondary structure that in effect goes against its amino acid composition. Improvement may be possible by considering higher order packing that can be provided by the knob-socket model. As an example, instead of strand predictions, a construct for tertiary packed sheets would potentially improve the accuracy by providing a strong differentiation over coils using the knob-socket model. While long range residue interactions have been integrated in a general sense into previous methods [26], [28], the knob-socket model provides constructs that can correlate specific residue patterns derived from packing interactions that identify secondary structure.

## Materials and Methods

### Data Set

The secondary structure data was derived from the ASTRAL SCOP 1.75 structure set [39] filtered at 95% sequence identity. This structure set consisted of 15,470 individual protein domains from the PDB [37] whose length range from 22 to 1,419 amino acids and total 2,751,815 amino acids. Besides the training set, we used two test sets. The first test set is the current release of SCOPe 2.03 data set [40] filtered at 30% sequence identity (ASTRAL30). In this ASTRAL30 set we included the domains that are not included in 1.75 version and only included in 2.03 version. The transmembrane proteins were also excluded and this gave 2,794 domains. The data set integrity was further tested by breaking down into the actual segments. When the structure has missing residues, the chain was split into separate sequences and omitted in this study if a chain is shorter than 25 residues. This produced 3,344 chains with 523,332 amino acids. The second test set was created from the targets used in CASP9 experiments in 2010 [41]. The CASP9 set includes 147 structures, and the same cleanup procedure produced 203 chains with 23,298 amino acids.

To compare the performance with TorusDBN [36] and PSIPRED [34], our method was trained with the older ASTRAL30 1.75 set [42], [43]. The training set did not contain any chains from either the ASTRAL30 and CASP9 test sets described, and so is properly jack-knifed with regards to the test data. TorusDBN and PSIPRED predictions were carried out locally using downloaded copies of the programs. For TorusDBN, the backbone-dbn-torus predictor program was used without any additional input. The prediction with PSIPRED was carried out with a BLAST [44], [45] search on two different databases. The NR is a full non-redundant sequence database with low-complexity regions filtered and the other nrPDB is the subset of sequences with determined structures in the PDB [37]. The BLAST search was also performed on a local computer with the downloaded program. Because of the large number of sequences in the NR database, the BLAST search took significantly longer than nrPDB. Multiple sequence alignments of the similar structures were obtained from the BLAST search with the nrPDB database for our MSA prediction. Also, in our prediction, the sequences in the ASTRAL30 and CASP9 were jack-knifed out of the nrPDB database. This insured that our MSA based predictions had no information from the native sequence.

### Notation

Let 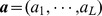 be an observed amino acid sequence, i.e., protein primary structure, where 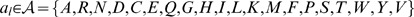 is a one-letter code denoting one of the 20 proteinogenic amino acids and *L* is the protein length. The secondary structure of a protein is the general form of its local segments, which we refer to as “block types”. [8] proposed the Dictionary of Protein Secondary Structure (DSSP) for protein secondary structure with single letter codes. Although generalizations may be desirable, we consider the following 4 block types (in italics) from the original 8 structures defined in DSSP (in parentheses):

- Helix “*H*”: 3_10_ helices (G), *α*-helices (H), or 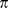-helices (I);
- Strand “*E*”: extended strands in parallel or anti-parallel *β*-sheets (E);
- Turn “*T*”: hydrogen bonded turns of length 3 or more amino acids (T);
- Coil “*C*”: *β*-bridge residues (B), bends (S), or random coils (C).

Let 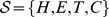 denote the set of block types. The secondary structure can be encoded in a convenient fashion by representing the structural types and segment length 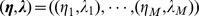, where 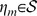 gives the secondary structure type in the *m*-th block and 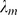 gives the length of that block. Note that 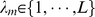 and 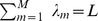. For example, Fig. 1 shows the representation of the secondary structure of the protein clathrin 1*c*9*l* [46].

In the case of secondary structure prediction, the quantities of interest are 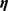 and 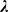 corresponding to the known amino acid sequence 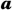, i.e., the type and length of each secondary structural segment. The cumulative length also contains the segment location information. Thus, mathematically, the problem is to infer the values of 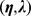 given the amino sequence 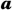.

### Sampling Model

We start by considering the joint distribution of the data 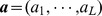 given the latent secondary structure, 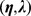. We write the joint probability mass function (p.m.f.) 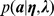 as a product over blocks:
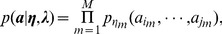
(1)where 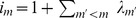 is the starting position of the *m*-th block, 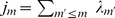 is its ending position, and 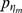 is one of 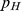, 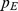, 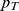, and 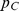 based on the value of 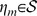. By grouping portions of the sequence into blocks, our method leverages the natural property that secondary structure states are necessarily formed by groups of residues. Our method thus captures the local context or environment around a residue that influences its secondary structure state, which aids prediction accuracy in all three states. As described below, 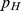, 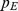, 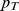, and 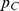 are designed to reflect the protein three-dimensional local structure at the molecular level. (See Fig. 2.)

We evaluate the sampling model for each block as the product of position-specific marginal or conditional distributions estimated from the PDB. At each position, the sampling model for a single amino acid 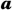 is of the form:
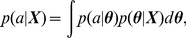
(2)where 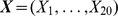 is the count vector for the number of times that each of the 20 amino acids is found in the training data (from the PDB) for the situation of interest. Specific situations are described in the following subsections and could be, for example, the start of a helical block or the third position in a strand with amino acids A then C proceeding it. When 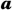 is viewed as a vector of length 20 with all zeros except a single 1, 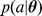 in (2) is a multinomial distribution with one trial and probability vector 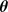. Assume the following Bayesian model: 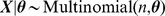 and 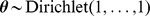, where 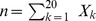. Due the conjugacy, the posterior distribution 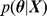 is 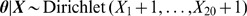. The integration of the product of 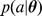 and 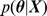 with respect to 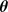 makes 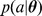 a Dirichlet-multinomial distribution. Because the number of trials is simply 1, evaluating 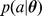 requires only that we divide one plus the number of times the amino acid 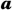 is present in the situation of interest in the training dataset by *n*+20.

#### Sampling Model for Helices

We propose that the sampling model for helices is defined by a product of four p.m.f.’s as follows:
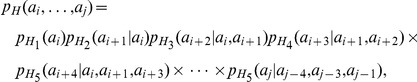
(3)where 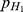 is a multinomial distribution with a category for each of the 20 amino acids, 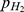 is a 20-dimensional multinomial distribution conditioned on the antecedent amino acid, 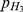 and 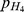 are multinomial distributions conditioned on the two previous amino acids, and 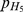 is a 20-dimensional multinomial distribution conditioned on the previous amino acid, the amino acid three positions back, and the amino acid four positions back. In the case of a short helical block, terms beyond the length of the helix are simply ignored (i.e., 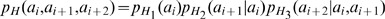 for a helix of three amino acids). This formulation for 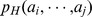 is tractable, yet still respects the biochemistry of helices, as shown in Fig. 2(a).

As previously explained, in our approach we evaluate the simpler p.m.f.’s in (3) based on training data. The 20-dimensional probability vector for 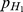 is taken to be the posterior mean from a Bayesian model assuming a multinomial sampling model and a non-informative Dirichlet prior with all hyperparameters equal to 1. The data for this estimation is obtained from the PDB by counting the number of helical blocks that start with each of the 20 amino acids. Similarly, since there are 20 amino acids on which to condition, there are 20 p.m.f.’s of type 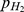 and 20×20 p.m.f.’s of type 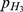. Likewise, since there are 20×20×20 = 8,000 combinations of three amino acids, there are 8,000 p.m.f.’s of type 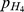. Again, these probability vectors for 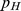 are calculated from all the sequences in the PDB and stored for evaluating the likelihood for a helical block as in (2).

As described to this point, the sampling model for helix is a “forward” model in which the contribution of each amino acid is conditioned on previous amino acids. An important aspect of the biochemistry of each block is the existence of capping signals: the preference, through side chain-backbone hydrogen bonds or hydrophobic interactions, for particular amino acids at the N- and C-terminals of a helix. Usually, the terminal end is the first and last 3 or 4 positions in a block [47], whose effect is reflected by the amino acid distribution which significantly differ from that of internal positions. These signals have been characterized experimentally in terms of their stabilizing effect in helical peptides [47].

Whereas the forward model captures the capping signal in the N-terminus, we also consider a “backward” model. The backward model is the exact opposite of the forward model. It is built sequentially by starting at the C-terminus of the block and working backwards to the front, each time conditioning on amino acids closer to the C-terminus. Apart from the direction, the form of the conditioning is the same as the forward model. Thus, the sampling model for the helical blocks is a mixture model, composed of the forward component and the backward component as follows:
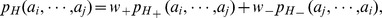
where 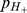 is the forward model defined in (3) and 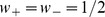. A mixture model is not the only way to handle both capping ends and, for example, a single unified model would also be valid. We do not expect a major difference in performance among models that account for capping. As such, we propose the two-component mixture model for ease of exposition.

#### Sampling Model for Strands

We propose that the sampling model for strands, with joint p.m.f. 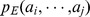, is defined by a product of six simpler p.m.f.’s 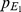, 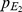, 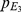, 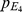, 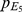, and 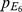 as follows:
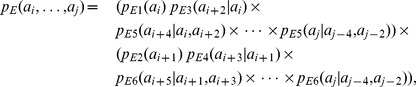
(4)


where 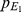 and 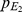 are a multinomial distribution with a category for each of the 20 amino acids, 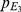 and 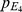 are 20-dimensional multinomial distributions conditioned on the value of the antecedent amino acid two positions back, etc. In the case of a short strand block, terms beyond the length of the strand are simply ignored (i.e., for a stand of length three, 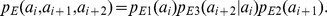 Again, this formulation 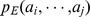 is tractable, yet still respects the biochemistry of strands, as shown in Fig. 2(b). Note that 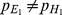, despite the fact that both are marginal multinomial distributions. Likewise, 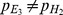, despite the fact that both are conditional multinomial distributions given an amino acid. In particular, 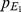, 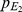, 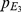, 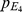, 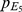, and 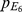 are estimated from PBD data involving strands, whereas 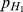, 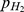, 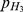, 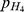, and 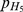 are estimated from PBD data involving helices, but the estimation strategy is the same.

#### Sampling Model for Coil

We propose a sampling model for coils as the product of p.m.f.’s as follows:
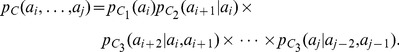
(5)


In the case of a short coil block, terms beyond the length of the coil are simply ignored (i.e., for a coil of length two, 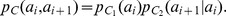 Again the formulation respects the biochemistry of coils as shown in Fig. 2(c) and the sampling models are estimated from the PDB.

#### Sampling Model for Turn

According to the turn structure as shown in Fig. 2(d), we propose that the sampling model for turns be defined by a product of p.m.f.’s, as follows:
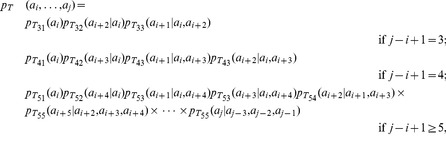
(6)where each conditional p.m.f. in the equation above is estimated based on the PDB data using hydrogen bonded turns of length 3, 4, and 5 or more amino acids, respectively.

### Prior Distribution

The model is completed by specifying the prior distribution, with p.m.f. 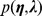. First, we make the p.m.f. equal zero if the biochemistry inherent in secondary structure is violated. Specifically it is zero if, for 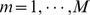, any of the following conditions are met:

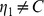
 or 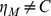 (i.e., if it does not start and end in coil)

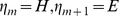
 (i.e., if helix is followed by strand)

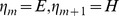
 (i.e., if stand is followed by helix)

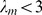
 and 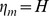 (i.e., if helix block is less than 3 positions)

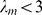
 and 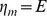 (i.e., if strand block is less than 3 positions)

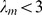
 and 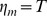 (i.e., if turn block is less than 3 positions).

The implies that helix, strand, coil, and turn blocks are at least 3, 3, 1, and 3 amino acids long, respectively. The first prior is a noninformative (NonInfo) prior and it provides equal weights to all the allowable secondary structures, that is,
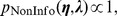
for all 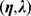 except the above listed conditions.

We also consider an informative prior distribution which incorporates multiple sequences alignment (MSA) information. For an observed amino acid sequence 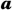, we first search for a set of proteins with similar amino acid sequences whose secondary structures is already known. The candidate database and the matching criterion are a modeling choice. For our analysis, we used a PSI-BLAST search of the nrPDB database. PSI-BLAST searches were performed on the local sever against the non-redundant protein sequence database with entries from GenPept, Swissprot, PIR, PDF, PDB and NCBI RefSeq, downloaded from NCBI website (ftp://ftp.ncbi.nih.gov/blast/db/nr.*). The low complexity sequence regions were filtered to avoid the artifactual hits. The structures with E-values better than 0.001 from the search were used in the alignments. Also, sequences already in the validation datasets (ASTRAL30 and CASP9) were excluded to insure that our MSA based predictions had no information from the native sequence. We build the prior distribution for 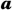 as the product of position-specific marginal distributions estimated from its corresponding alignment outputs. Let
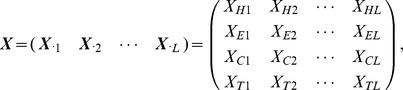
where 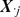 is the count vector for the number of times that each of the four secondary structure types is found in the *j*-th position of the alignment output. Assume the following Bayesian model:
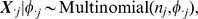
and
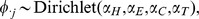
where 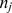 is the number of aligned sequences minus the number of times that gap is found in the *j*-th position. Due to the conjugacy, the posterior distribution is
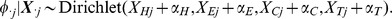



We suggest default values of 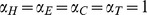. Let
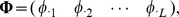
then we assume the secondary structure sequence follows a product of *L* p.m.f.’s, i.e.,
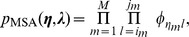
where *l* indexes the position, 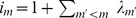 is the starting position of the *m*-th block, and 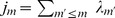 is its ending position.

### MCMC Algorithm

Our goal is to make inference on the secondary structure 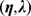 given the amino acid sequence 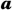. We use Markov chain Monte Carlo (MCMC) methods to sample from the posterior distribution:
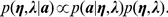
(7)


We update 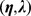 using a Metropolis algorithm. The factorization in (1) allows Hastings ratios to be evaluated locally with respect to the affected segments [48]. We note that this algorithm is sufficient to guarantee ergodicity for our model. In this algorithm, a new candidate 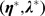 is generated according to the following scheme:

- Switch the type of a randomly chosen block: Randomly choose a number 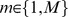 and change the new *m*-th block type to 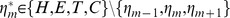 with equal probability. Leave all other block types and lengths unchanged.
- Change the position of boundary between two blocks: Randomly choose a number 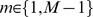 and draw the new *m*-th block length 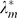 from 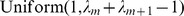 and hence the new 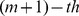 block length 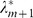 equals to 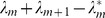. Leave all block types and other lengths unchanged.
- Split a block into two adjacent blocks: Randomly choose a number 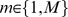. Make space for a new block to be placed between blocks *m* and 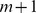 as follows. Let 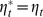 and 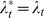 for 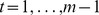, and let 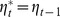 and 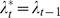 for 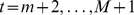. Let 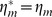. What remain to define are values for 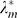, 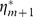, and 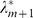. Assign the new (*m*+1)-th block type to 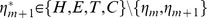 with equal probability. Draw the new *m*-th block length 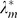 from 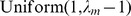 and hence the new (*m*+1)-th block length 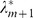 equals to 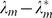.
- Merge two adjacent blocks into one block: Randomly choose a number 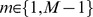. Let 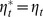 and 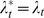 for 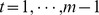, and let 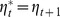 and 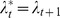 for 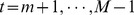. Finally, let 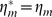 and 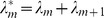.

The Hastings ratio can be written as:
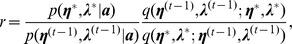
where 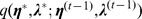 is the proposal density, the density for proposing a move to 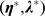 given the previous state 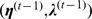 and 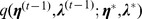 is the reverse case. The move is accepted 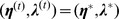 with the probability min(1, *r*), otherwise, the move is rejected and 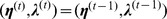.

For the results presented in this paper, 1,000,000 MCMC proposal were obtained (each time randomly selecting among the four proposal schemes described earlier). About half of the proposals lead to valid secondary structures. (For example, proposing to switch a block to helix is not valid if the block is already adjacent to an helix block.) Among the valid proposals, about 20% were accepted. The first 10,000 samples were discarded for burnin. MCMC convergence can be assessed by comparing the stability of the marginal probabilities of the states at each position across independent MCMC runs with different starting secondary structure states.

### Posterior Estimation

The goal is to infer the secondary structure 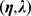. We considered two ways to summarize the posterior distribution to yield a point estimate. Among all samples obtained by the MCMC algorithm, choose the 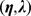 that maximizes the posterior probability 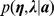:
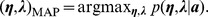



We name this estimate as maximum *a posteriori* (MAP) estimate.

To describe the second posterior estimation method, it is convenient to introduce the *linear sequence* parameterization that encodes the secondary structure using a vector 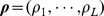, where 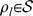 indicates the secondary structure at position *l*. This parameterization encodes the same information as the original parameterization 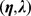. We construct the estimate by selecting the most likely block type for each position:
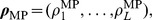
where 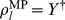 if 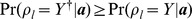 for 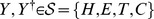 and 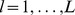. We call estimates obtained in this manner marginal probability (MP) estimates.

## Conclusions

A statistical model for knob-socket packing [30], [31] between residues has been developed for prediction of protein secondary structure. The unique feature of this approach is that the knob-socket model provides constructs for the direct inclusion and prediction of the secondary states of coil and turn (Fig. 2(c) and (d), respectively). Other secondary structure prediction methods do not make direct prediction of coil structure and essentially apply indirect identification of coil residues as neither helix and sheet. We assess our method’s Q3 prediction accuracy on 2 test sets and compare results with those obtained with the benchmark method PSIPRED [12]. From an investigation of the accuracy of prediction for each state, we found improved predictions adding context in terms of blocks of amino acids; however, our basic model over predicts the coil state. We show how incorporation of multiple sequence alignment data, similarly in spirit to PSIPRED, provides balance and improves prediction accuracy. Indeed, our method achieves slightly less accurate predictions than does PSIPRED on one test set, and almost reaches 90% on the other. Our results reinforce the general concept that more context is necessary to understand the environment that induces secondary structure.

Our work takes the initial step to enable Bayesian method to infer the secondary structure of proteins and serves as a call for participation. Many interesting and important directions are worth exploring. For example, our work is limited in the sense that only considers local dependency. We are exploring several ways of incorporating non-local information in future work. This may be especially beneficially improving strand predictions. Another interesting line of research is how to borrow information across probability vectors in the sampling models to improve the algorithm performance.
